# Fever of unknown origin in adults: diagnostic spectrum, the contribution of FDG-PET/CT and targeted sequencing, and spontaneous resolution

**DOI:** 10.3389/fimmu.2026.1899032

**Published:** 2026-10-23

**Authors:** Masataka Umeda, Lamiaa Mohamed, Kazusato Hara, Mizuna Otsuka, Shota Kurushima, Tomohisa Uchida, Toru Michitsuji, Yoshika Tsuji, Yushiro Endo, Ayuko Takatani, Shoichi Fukui, Remi Sumiyoshi, Tomohiro Koga, Shin-ya Kawashiri, Naoki Iwamoto, Takashi Igawa, Mami Tamai, Tomoki Origuchi, Hiroo Izumino, Takahiro Maeda, Koya Ariyoshi, Atsushi Kawakami

**Affiliations:** 1 Department of Immunology and Rheumatology, Nagasaki University Graduate School of Biomedical Sciences, Nagasaki, Japan; 2 Department of General Medicine, Nagasaki University Graduate School of Biomedical Sciences, Nagasaki, Japan; 3 Nagasaki Medical Education Center (N-MEC), Nagasaki University Hospital, Nagasaki, Japan; 4 Leading Medical Research Core Unit, Nagasaki University Graduate School of Biomedical Science, Nagasaki, Japan; 5 Department of Rheumatology, Saiseikai Nagasaki Hospital, Nagasaki, Japan; 6 Acute and Critical Care Center, Nagasaki University Hospital, Nagasaki, Japan; 7 Department of Clinical Medicine, Institute of Tropical Medicine, Nagasaki University, Nagasaki, Japan

**Keywords:** autoinflammatory diseases, diagnosis, FDG-PET/CT, fever of unknown origin, next-generation sequencing

## Abstract

The diagnostic paradigm for fever of unknown origin (FUO) has shifted with the advent of functional imaging and genomic technologies. This study evaluated the clinical impact of targeted next-generation sequencing (NGS) and 18-FDG-PET/CT in a single-center FUO cohort, while examining clinical features associated with spontaneous resolution. Among 116 patients analyzed, definitive diagnoses were established in 75.9%. In a selected subgroup with strong clinical suspicion of autoinflammatory disease, targeted NGS identified candidate variants in 88.9%, supporting clinico-genomic diagnostic confirmation in integration with clinical criteria. In a descriptive series of 15 cases evaluated by FDG-PET/CT at attending physicians’ discretion, imaging contributed to direct diagnosis in 33.3%. Notably, 25 of 28 undiagnosed patients experienced spontaneous resolution. Higher hemoglobin, higher albumin, absence of renal dysfunction, and a preserved appetite were identified as exploratory clinical markers associated with spontaneous remission; however, the association for appetite was attenuated in multivariable models adjusted for baseline status. While advanced technologies are valuable diagnostic tools in selected high-pre-test probability settings, a substantial proportion of undiagnosed FUO cases resolve spontaneously. A preserved appetite, alongside systemic baseline stability, may serve as an exploratory clinical surrogate supporting a cautious “watchful waiting” strategy in selected patients.

## Introduction

Fever of unknown origin (FUO) remains one of the most diagnostically challenging conditions encountered in clinical practice and continues to pose a substantial diagnostic challenge. The most widely accepted definition of FUO is based on the revised criteria proposed by Durack and Street in 1991, which characterize FUO as a body temperature of ≥38.3 °C on several occasions lasting for at least three weeks, with no established diagnosis after three days of hospital investigation or three outpatient visits (1). Traditionally, infections, malignancies, and inflammatory diseases—including connective tissue disorders and vasculitides—are considered the three principal etiologic categories of FUO (2). Advances in diagnostic technologies, such as high-resolution imaging and ultrasound-guided tissue sampling, have improved diagnostic yield over time. Interestingly, these advances have coincided with a reported decline in the proportion of FUO cases attributed to infections and malignancies, while the proportion of cases that remain undiagnosed has increased (2). This paradox highlights the growing complexity of FUO in contemporary clinical practice. Comprehensive patient evaluation—including detailed history-taking (e.g., recent travel, animal exposure, and medication use), physical examination, laboratory testing, microbiological cultures, histopathological analysis, and immunological assessments—remains the cornerstone of the FUO workup (3). In recent years, the addition of 18F-fluorodeoxyglucose positron emission tomography combined with computed tomography (FDG-PET/CT) has been shown to offer diagnostic clues in a subset of patients with otherwise elusive diagnoses (4). Moreover, molecular diagnostic approaches, such as broad-range polymerase chain reaction (PCR) targeting bacterial 16S and fungal 18S ribosomal RNA genes, and next-generation sequencing (NGS), have emerged as powerful tools to identify diverse pathogens rapidly and comprehensively, thereby enabling more targeted therapeutic strategies in the context of FUO (5). These advances have broadened the range of diagnostic tools available for FUO. At our institution, targeted next-generation sequencing (NGS) has been integrated into routine clinical practice to facilitate the genetic diagnosis of autoinflammatory diseases, including familial Mediterranean fever (FMF) (6). However, the integration of these advanced genomic technologies into the diagnostic algorithm for FUO remains a subject of ongoing debate. In particular, their real-world impact on diagnostic pathways and patient outcomes has not been fully elucidated. In addition, the clinical course of patients with undiagnosed FUO remains insufficiently characterized. While some patients ultimately receive a diagnosis, others experience spontaneous resolution without specific treatment. Identifying predictors of such outcomes may help guide clinical decision-making and avoid unnecessary invasive investigations. In this study, we provide a descriptive analysis of the clinical course, diagnostic process, and final diagnoses of patients with FUO after the introduction of NGS-based autoinflammatory gene testing at our institution, with a particular focus on the diagnostic contribution of advanced technologies and factors associated with spontaneous resolution.

## Materials and methods

### Patients

We conducted a retrospective review of patients admitted to the Departments of General Medicine, Infectious Diseases, and Rheumatology at Nagasaki University Hospital between March 2019 and February 2023. Eligible patients met the classical criteria for fever of unknown origin (FUO) as defined by Durack and Street in 1991—namely, a fever ≥38.3 °C on several occasions lasting for at least three weeks, with no definitive diagnosis after three days of inpatient evaluation or three outpatient visits. Spontaneous resolution was defined as maintaining normal body temperature (<37.0 °C) without specific anti-inflammatory or immunosuppressive medications for at least 1 year, or the absence of re-consultation for recurrent fever at our hospital. This study period corresponds to the four years following the implementation of NGS–based genetic testing for autoinflammatory diseases at our institution. 18F-FDG-PET/CT was not protocol-driven; it was performed when the attending physician judged it necessary, generally after the initial evaluation had not localized a source of fever. A scan was considered contributory when its findings established the final diagnosis or directly led to the procedure that did. Patient information was extracted from electronic medical records.

### Targeted next-generation sequencing

Targeted NGS was not applied systematically to the cohort. It was performed when the attending physician judged, on the basis of the clinical course, that a monogenic autoinflammatory disease was a plausible cause of the fever. The patients tested therefore constitute a clinically selected subgroup rather than a consecutive series. The details of our NGS protocol for autoinflammatory gene analysis have been described previously (6) and provided in **Supplementary Material**. Variant classifications are provided in **Supplementary Table S1**, and the clinical criteria used for final diagnostic confirmation are described in the **Supplementary Material**.

### Ethics approval and consent to participate

This study was performed in accordance with the Declaration of Helsinki and was approved by the institutional review board of Nagasaki University Hospital (Approval no. 222112112). Participants currently receiving treatment were provided with detailed information about the study’s purpose, procedures, risks, and the voluntary nature of participation, and verbal consent was obtained. For all other patients, an opt-out option was provided through a publicly available information document, allowing them to decline participation if desired.

### Data analysis

Kaplan-Meier curve was generated using GraphPad Prism, version 10.5.0 (GraphPad Software, San Diego, CA, USA). The statistical analyses were performed using JMP Pro 17.0 Statistical Software (SAS Institute, Cary, NC, USA). Continuous variables were compared using Wilcoxon’s rank sum test, while categorical variables were analyzed with Fisher’s exact test. Associations with spontaneous resolution of fever were assessed by logistic regression, with odds ratios, 95% confidence intervals and p values. Dietary intake was adjusted separately for each of these variables — CRP, albumin, haemoglobin and abnormal renal function — in a series of two-covariate models. To ensure analytical rigor and eliminate potential outcome heterogeneity in the comparator group, all logistic regression analyses were conducted both in the full cohort (n=116) and in a restricted sensitivity cohort (n=113), which excluded the 3 patients who remained undiagnosed without spontaneous resolution. Statistical significance for all tests was defined as a two-tailed p-value of less than 0.05.

## Results

### Overall diagnostic distribution of FUO in the era of genome analysis

Among the 2,099 total admissions during the study period, 116 patients (5.5%) met the criteria for FUO. The demographic characteristics of patients with FUO are summarized in **Table 1**. The median age was 66 years (interquartile range, 48.3–78), and 56 patients (48.3%) were male. The median interval from fever onset to hospitalization was 28 days (interquartile range, 17–55.8). The final diagnoses identified during hospitalization are summarized in **Table 2**. Diagnoses included autoimmune and autoinflammatory diseases in 44 (37.9%), infectious diseases in 17 cases (14.7%), malignancies in 18 (15.5%), and other miscellaneous causes in 9 (7.8%). Notably, 28 patients (24.1%) remained undiagnosed despite extensive investigation, of whom 25 (89.3%) eventually experienced spontaneous resolution of fever.

**Table 1:** Demographic characteristics of patients with fever of unknown origin (n=116).

Characteristic	Overall Cohort (n=116)
Age (years)	66 (48.3-78)
Gender (Male)	56/116 (48.3%)
Time from onset of fever to hospitalization (days)	28 (17-55.8)
White blood cell(/µl)	8800 (5925-11150)
Neutrophil (/µl)	6140 (3892.5-8692.5)
Lymphocyte (/µl)	1125(685-1527.5)
Hemoglobin (g/dl)	10.9 (9.1-12.2)
Platelet (/10^4^ μl)	23.4 (14.3-36.9)
CRP (mg/dl)	6.0 (1.85-11,1)
Total protein (g/dl)	6.9 (6.1-7.5)
Albumin (g/dl)	2.8 (2.3-3.4)
Abnormal renal function	34/116, (29.3%)
Abnormal liver function	80/116, (69.0%)
Dietary intake (%)	70.5 (34-93.8)

Values are presented as median (quartile) or number (%). Abnormal renal function is defined as an elevated serum creatinine level. Abnormal liver function is defined as elevations in AST, ALT, γ-GTP, or ALP. Dietary intake represents the average percentage of food intake during the first 3 days of hospitalization.

**Table 2:** Final diagnoses of patients with fever of unknown origin (N = 116).

Category	Diagnosis	n
Autoimmune/Autoinflammatory (n = 44, 37.9%)	Microscopic polyangiitis	6
	Still’s disease	5
	Giant cell arteritis	5
	Idiopathic multicentric Castleman’s disease	4
	TAFRO syndrome	4
	Familial Mediterranean fever	3
	Polyarteritis nodosa	3
	VEXAS syndrome	2
	Granulomatosis with polyangiitis	2
	Systemic lupus erythematosus	2
	Human adjuvant disease	1
	Takayasu arteritis	1
	RS3PE syndrome	1
	Mixed connective tissue disease	1
	Behçet’s disease + Familial Mediterranean fever	1
	Blau syndrome	1
	Ulcerative colitis	1
	Hashimoto’s encephalopathy	1
Infectious (n = 17, 14.7%)	HIV infection†	3
	Pyogenic spondylitis	2
	Aseptic meningitis	2
	Mycoplasma infection	2
	Infectious mononucleosis	1
	Aortic aneurysm infection	1
	VP shunt infection	1
	Bronchopneumonia	1
	Cryptococcal meningitis	1
	Urinary tract infection	1
	Splenic abscess	1
	Infective endocarditis	1
Malignancy/Hematologic (n = 18, 15.5%)	Malignant lymphoma‡	15
	Advanced gastric cancer	1
	Bladder cancer	1
	Leukemia	1
Other (n = 9, 7.8%)	Dialysis-related fever	2
	Functional fever	2
	Erythema nodosum	1
	Hemangiomyolipoma	1
	Eosinophilic myocarditis	1
	Renal infarction	1
	MTX-related lymphoproliferative disorder	1
Undiagnosed (n = 28, 24.1%)	Spontaneous remission	25
	Suspected autoinflammatory disease	2
	Alveolar hemorrhage, suspected vasculitis§	1

†HIV infection included one case complicated by neurosyphilis.

‡Malignant lymphoma included two cases of intravascular large B-cell lymphoma.

§The patient with alveolar hemorrhage and suspected vasculitis died after transfer to another hospital at the patient’s request, not during the initial hospitalization.

### Impact of targeted NGS incorporate with clinical findings on the identification of autoinflammatory diseases

To refine the diagnostic utility for elusive cases, targeted NGS was performed in selected patients in whom autoinflammatory conditions were clinically suspected. This approach identified genetic variants in 8 out of 9 patients (88.9%; **Table 3**). Although some identified variants were of uncertain significance or potentially benign (**Supplementary Table S1**), the final clinical diagnoses were established by integrating these NGS results with each patient’s clinical course and treatment response. Consequently, definitive diagnoses—such as familial Mediterranean fever, VEXAS syndrome, and Blau syndrome—were made. One patient with an MEFV variant experienced spontaneous resolution, while another case with no identifiable genetic abnormalities remained undiagnosed.

**Table 3:** Results of targeted next-generation sequencing and final clinical diagnoses in patients with fever of unknown origin.

Age (years)	Gender	Genetic variants	Final diagnosis
58	Female	MEFV: p.Arg202Gln (heterozygous)	familial Mediterranean fever
27	Female	MEFV: p.[Leu110Pro; Glu148Gln] (heterozygous)	familial Mediterranean fever
35	Male	MEFV: p.Ser503Cys	familial Mediterranean fever
69	Female	MEFV: p.[Leu110Pro; Glu148Gln] (heterozygous)	Familial Mediterranean fever/Bechet’s disease
73	Male	UBA1: p.Met41Thr (hemizygous)	VEXAS syndrome (diagnosed post mortem)
70	Male	UBA1: p.Met41Val (hemizygous), MEFV: p.[Glu148Gln; Pro369Ser; Arg408Gln] (heterozygous)	VEXAS syndrome
42	Female	NOD2: p.[R311W; R471C] (heterozygous)	Blau syndrome
19	Female	MEFV: p.[Leu110Pro; Glu148Gln] (heterozygous)	Undiagnosed (Resolved spontaneously)
62	Female	No abnormality	Undiagnosed

Final clinical diagnoses were established by integrating genetic findings with the patient’s clinical course and treatment response. Genetic variants are described according to HGVS(Human Genome Variation Society) nomenclature. Detailed Genetic Profiles is described in **Supplementary Table S1**.

### Diagnostic contribution of FDG-PET/CT to comprehensive evaluation

The utility of FDG-PET/CT was performed in 15 of the 116 patients (12.9%) (**Table 4**). Imaging was undertaken a median of 34 days after the onset of fever (interquartile range, 22–71 days) and provided decisive diagnostic clues in 33.3% (5 of 15) of the cases in which it was performed, particularly aiding in the identification of malignant lymphoma and large vessel vasculitides at the discretion of the attending physician. Of the patients who remained undiagnosed despite the comprehensive evaluation including FDG-PET/CT imaging, three individuals experienced spontaneous resolution of their fever.

**Table 4:** Diagnostic utility of comprehensive clinical assessment in 15 patients who underwent FDG-PET/CT.

Age (years)	Gender	Days from fever onset to FDG-PET/CT	Diagnostic Contribution of FDG-PET/CT	Final diagnosis
59	Male	295	+	Malignant lymphoma
50	Male	58	+	Angiomyolipoma
32	Female	35	+	Takayasu arteritis
67	Female	19	+	Giant cell arteritis
66	Male	12	+	Fasciitis of the thigh
81	Female	63	–	Bladder cancer
79	Female	17	–	Systemic lupus erythematosus
89	Female	88	–	Still’s disease
74	Female	25	–	Microscopic polyangiitis
81	Female	31	–	Microscopic polyangiitis
75	Female	12	–	Dialysis related fever
68	Male	79	–	Undiagnosed (Suspected autoimmune disease)
79	Female	31	–	Undiagnosed (Resolved spontaneously)
69	Male	34	–	Undiagnosed (Resolved spontaneously)
19	Female	110	–	Undiagnosed (Resolved spontaneously)

FDG-PET/CT, 18F-fluorodeoxyglucose positron emission tomography combined with computed tomography.

### Duration from fever onset to spontaneous resolution in patients with undiagnosed fever of unknown origin

Of the 28 patients who remained undiagnosed despite extensive clinical evaluation, 25 patients (89.3%) achieved spontaneous resolution of fever without specific treatment over a median follow-up duration of 233 days (interquartile range, 67–1,664 days). The time course of these 25 cases is illustrated in **Figure 1**. The cumulative resolution rate, analyzed via a reverse Kaplan-Meier curve, showed a median duration from fever onset to resolution of 41 days (interquartile range, 32–56.5 days). Notably, approximately 80% of these individuals recovered within 60 days, and all 25 cases reached complete defervescence by day 250, suggesting a favorable prognosis for the majority of undiagnosed FUO cases in this cohort.

**Figure 1:**
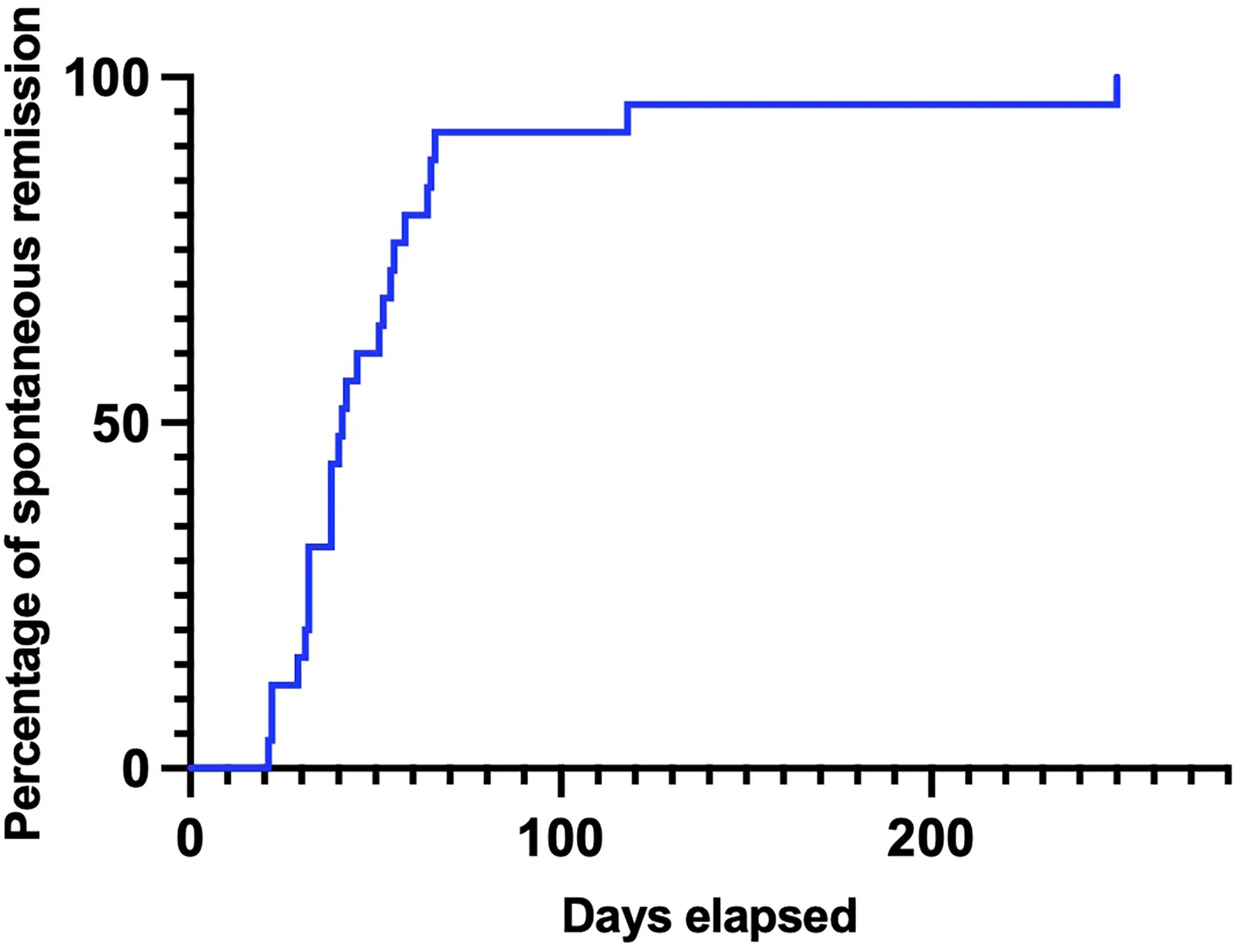
Time to spontaneous resolution in patients with undiagnosed fever of unknown origin. Among the 28 patients whose diagnoses were not established, 25 (89.3%) experienced spontaneous resolution of fever. The cumulative resolution rate for these 25 cases is shown using a reverse Kaplan-Meier curve. The median time to resolution was 41 days (interquartile range, 32–56.5 days).

### Association between preserved appetite and spontaneous fever resolution

To facilitate clinical decision-making, we investigated the prognostic factors associated with spontaneous resolution of fever. Predicting which patients will recover without specific intervention is crucial in the management of FUO. Univariate logistic regression analysis (**Table 5**) identified several clinical and laboratory parameters at admission that significantly predicted spontaneous resolution. Higher hemoglobin levels (OR 1.26, 95% CI 1.04–1.56; p = 0.02) and higher albumin levels (OR 1.96, 95% CI 1.02–3.77; p = 0.04), along with the absence of renal dysfunction (OR 0.16, 95% CI 0.03–0.72; p = 0.004), were positive predictors of a favorable outcome. Notably, a preserved appetite—quantified as the average percentage of dietary intake during the first 3 days of hospitalization—was significantly associated with an increased likelihood of spontaneous resolution (OR 1.02 per 1% increase, 95% CI 1.001–1.03; p = 0.03). Because the number of events limited the number of covariates that could be entered simultaneously, dietary intake was adjusted individually for potential confounders (**Supplementary Table S2**). The odds ratio for dietary intake was 1.016 per 1% increase (95% CI 1.001–1.031; p = 0.039) before adjustment and was unchanged after adjustment for CRP (1.016, 95% CI 1.001–1.031; p = 0.040), but was attenuated to 1.012–1.013 and no longer reached statistical significance after adjustment for albumin (p = 0.111), haemoglobin (p = 0.137) or abnormal renal function (p = 0.119). The direction of the association was the same in every model. The same pattern was obtained when the 3 patients who remained undiagnosed without spontaneous resolution were excluded, so that the comparator consisted only of patients in whom a diagnosis had been established (n = 113; **Supplementary Table S2**).

**Table 5:** Prognostic factors for spontaneous resolution in patients with fever of unknown origin.

Factors	With diagnosis and/or no spontaneous remission (n=91)	Without diagnosis, but resolved spontaneously (n=25)	Odds ratio (95% CI)	p-value
Age (years)	66 (50-78)	63 (40-70)	0.98 (0.96-1.00)	0.11
Gender (Male)	42/91 (46.1%)	14/25 (56.0%)	0.5 (0.28-1.64)	0.38
Days from fever to hospitalization	28 (17-57)	28 (16.5-51)	1.0 (0.99-1.00)	0.28
WBC (1000/µl)	9.2 (6.3-11.5)	7.0 (4.6-9.5)	0.89 (0.79-0.997)	0.04
Neutrophil (1000/µl)	6.6 (4.2-9.2)	4.7 (2.5-7.6)	0.88 (0.88-1.005)	0.06
Lymphocyte (1000/µl)	1.2 (0.6-1.5)	1.1 (0.8-1.6)	1.14 (0.63-2.07)	0.67
Hb (g/dl)	10.4 (8.8-11.9)	12.1 (10.5-12.8)	1.26 (1.04-1.56)	0.02
PLT (/104 μl)	22.7 (13.5-34.9)	29.3 (20.4-40.8)	1.03 (1.0-1.05)	0.05
CRP (mg/dl)	6.49 (2.1-10.9)	3.74 (1.4-13.4)	0.99 (0.94-1.06)	0.83
TP (g/dl)	6.7 (6-7.5)	7.1 (6.6-7.6)	1.56 (0.94-2.60)	0.08
ALB (g/dl)	2.7 (2.3-3.3)	3.3 (2.6-3.6)	1.96 (1.02-3.77)	0.04
Abnormal renal function (yes)	32/91 (35.2%)	2/25 (8.0%)	0.16 (0.03-0.72)	0.004
Abnormal liver function (yes)	65/91 (71.4%)	15/25 (60.0%)	0.6 (0.24-1.51)	0.28
Dietary intake (%)	68 (30-89)	80 (57-100)	1.02 (1.001-1.03)	0.03

Data are presented as median (interquartile range) or number (%). CI, confidence interval; WBC, white blood cell; Hb, hemoglobin; ALB, albumin. Abnormal renal function was defined as an elevated serum creatinine level above the upper limit of normal; abnormal liver function was defined as an elevation of aspartate aminotransferase (AST), alanine aminotransferase (ALT), γ-glutamyl transpeptidase (γGTP), or alkaline phosphatase (ALP). Dietary intake was calculated as the average of the percentage of food intake provided during the first 3 days of hospitalization, serving as a clinical indicator of preserved appetite.

## Discussion

The diagnostic approach to FUO has been fundamentally transformed by the advent of functional imaging and molecular genetics. In the present study, we demonstrated that the integration of targeted NGS and FDG-PET/CT allows for a more granular characterization of febrile etiologies, particularly in identifying rare autoinflammatory conditions and occult malignancies. However, our findings also highlight a critical, often overlooked aspect of FUO management: a significant proportion of undiagnosed cases follow a benign, self-limiting course, suggesting that “watchful waiting” remains a viable and evidence-based strategy when systemic stability is maintained.

The emergence of adult-onset autoinflammatory diseases, such as VEXAS syndrome and late-onset FMF, has shifted the FUO paradigm (7, 8). In our selected subgroup with strong clinical suspicion, targeted NGS identified candidate or pathognomonic variants in 88.9% of cases, highlighting its utility as a valuable diagnostic adjunct when applied to patients with high pre-test probability. Yet, the detection of a genetic variant requires cautious interpretation and should not be viewed in isolation (9). As seen in our case involving an MEFV variant that resolved without therapy, the presence of a genotype does not always dictate a persistent or severe phenotype. This necessitates a “clinico-genomic” integration, where the decision to initiate potentially toxic treatments (e.g., immunosuppressants) must be balanced against the patient’s actual clinical trajectory.

The diagnostic utility of FDG-PET/CT in FUO has been reported to be approximately 50-60% in several series (4, 10). Recent evidence emphasizes that early implementation of FDG-PET/CT can shorten the time to diagnosis and reduce the need for more invasive procedures (11). In our cohort, while PET/CT provided decisive clues in 33.3% of cases, its value extended beyond direct diagnosis, even though these data reflect a limited patient population selected at the discretion of attending physicians. A negative PET/CT finding often coincided with a favorable prognosis, serving as a powerful “negative predictor” (12) for aggressive diseases such as intravascular lymphoma or large-vessel vasculitis. This aligns with the concept that when sophisticated imaging fails to detect a localized inflammatory or neoplastic focus, the likelihood of an imminent catastrophic outcome is low (13, 14), thereby supporting a more conservative management approach.

The most striking finding of our analysis is that 89.3% of undiagnosed patients achieved spontaneous resolution, a rate that echoes findings from other large cohorts, such as a recent study in China where 41 out of 119 FUO patients experienced spontaneous remission (15). Building on this observation, we further identified preserved appetite as a clinically relevant marker of spontaneous resolution. While conventional inflammatory markers like CRP reflect the intensity of the inflammatory response, they do not necessarily reflect the patient’s functional reserve. In our multivariable adjusted models, the association between dietary intake and spontaneous resolution persisted after adjustment for CRP, but was attenuated and lost statistical significance when adjusted for albumin, hemoglobin, or renal function. These analyses indicate that a preserved appetite should be interpreted as an exploratory, hypothesis-generating clinical marker that is closely intertwined with underlying inflammatory burden and systemic baseline status (reflected by hemoglobin, albumin, and renal status), rather than an independent predictor. Although serum cytokines were not directly measured in this study, experimental and clinical studies have established that systemic inflammatory and anorexigenic cytokines (e.g., IL-1, IL-6, and TNF-α) induce acute appetite suppression and catabolic stress (16, 17). In light of this physiological framework, the maintenance of oral intake shortly after admission is best viewed as a holistic clinical surrogate for lower overall catabolic/inflammatory burden and the preservation of systemic homeostasis. Consequently, our findings suggest that a “watchful waiting” strategy is not merely a fallback for diagnostic failure, but a cautious, candidate clinical approach in selected, clinically stable patients who maintain good performance status, normal organ function, and preserved appetite. This approach reduces the risk of complications from empirical treatments, such as steroids or antibiotics, which can mask underlying pathologies or cause independent harm.

Several limitations of this study should be acknowledged. First, because this was a retrospective study conducted at a single tertiary center, referral and admission biases may limit the generalizability of our findings to primary or secondary care settings. Second, the sample size—particularly for the subgroup of patients who remained undiagnosed but experienced spontaneous resolution was relatively small (n=25). Third, pre-admission dietary intake was not recorded; thus, true baseline appetite could not be evaluated, and time-dependent bias or pre-hospitalization fluctuations in appetite cannot be ruled out. Future prospective, multicenter studies with larger patient cohorts are warranted to validate these findings through multivariable adjusted models.

In conclusion, the modern management of FUO requires a dual-track expertise: the proficiency to utilize cutting-edge technologies like NGS and FDG-PET/CT to identify treatable conditions early, and the clinical wisdom to recognize when a patient’s stable systemic status and preserved appetite signal an impending spontaneous resolution. By integrating these strategies, clinicians can provide personalized care that is both technologically advanced and humanistically prudent, avoiding the pitfalls of over-investigation while ensuring that truly critical diagnoses are not missed.

## Data Availability

The raw data supporting the conclusions of this article will be made available by the authors, without undue reservation.
