# Supplementary material for: Fever of unknown origin in adults: diagnostic spectrum, the contribution of FDG-PET/CT and targeted sequencing, and spontaneous resolution
Source: Front Immunol. 2026 Oct 23;17:1899032. doi: 10.3389/fimmu.2026.1899032 (PMC13739789; doi:10.3389/fimmu.2026.1899032)
Supplement: Supplementary file 1 [file SupplementaryFile1.docx]

Supplementary Material

**Fever of Unknown Origin in Adults: Diagnostic Spectrum, the Contribution of FDG-PET/CT and Targeted Sequencing, and Spontaneous Resolution**

Masataka Umeda, Lamiaa Mohamed, Kazusato Hara, Mizuna Otsuka, Shota Kurushima, Tomohisa Uchida, Toru Michitsuji, Yoshika Tsuji, Yushiro Endo, Ayuko Takatani, Shoichi Fukui, Remi Sumiyoshi, Tomohiro Koga, Shin-ya Kawashiri, Naoki Iwamoto, Takashi Igawa, Mami Tamai, Tomoki Origuchi, Hiroo Izumino, Takahiro Maeda, Koya Ariyoshi, and Atsushi Kawakami

Supplementary Methods

**Detailed Targeted Next-Generation Sequencing (NGS) Protocol and Variant Classification**

Targeted next-generation sequencing (NGS) was performed using a custom-designed gene panel targeting autoinflammatory diseases. Two versions of the panel were used during the study period: a 31-gene panel until 2021, and an updated 32-gene panel from 2022 onward, which specifically incorporated UBA1 (NLRP3, MVK, PSTPIP1, MEFV, TNFRSF1A, NOD2, IL1RN, NLRP12, NLRC4, PLCG2, HMOX1, TNFAIP3, ADA2, COPA, PSMB8, PSMA3, PSMB4, PSMB9, POMP, OTULIN, RNF31, RBCK1, LPIN2, IL36RN, CARD14, TREX1, RNASEH2A, RNASEH2B, SAMHD1, ADAR, IFIH1, and UBA1). Neither whole-genome sequencing (WGS) nor whole-exome sequencing (WES) was performed. Genomic DNA was extracted from peripheral blood leukocytes. Libraries were prepared using the Ion AmpliSeq Library Kit Plus and sequenced on the Ion PGM system using Ion 318 Chips and the Ion PGM Hi-Q View Sequencing Kit (Thermo Fisher Scientific, Waltham, MA, USA). Reads were aligned to the GRCh38 reference genome, and variant calling and annotation were executed using Ion Reporter software.

Germline versus somatic origin was determined based on variant allele fraction (VAF) and chromosomal location. Because UBA1 is an X-linked gene and both affected patients were male, a germline mutation would yield a VAF of approximately 100%. The presence of a wild-type allele in peripheral blood indicated somatic mosaicism. Although non-hematopoietic tissues (e.g., buccal mucosa or nails) were not evaluated, both identified variants occurred at the established Met41 hotspot, confirming the diagnosis of VEXAS syndrome (OMIM 301054).

For validation or specific candidate testing, Sanger sequencing was performed. In a 73-year-old male patient with VEXAS syndrome, in addition of targeted NGS analysis for 31-gene panel, Sanger sequencing identified UBA1 c.121A>C (p.Met41Leu, NM_003334.4); although precise sequencing depth and VAF cannot be directly calculated from Sanger electropherograms, the mutant allele fraction was semi-quantitatively estimated at approximately 50% based on peak height ratios. In a 70-year-old male patient with VEXAS syndrome, targeted NGS identified UBA1 c.121A>G (p.Met41Val, NM_003334.4) with a VAF of 80.7% (690 mutant reads out of a total depth of 855×; 365 forward and 325 reverse reads, showing no strand bias). Variant Classification: Identified genetic variants were annotated using standard genomic databases (ClinVar).

**Diagnostic references applied to the autoinflammatory diagnoses**

The clinical diagnosis of familial Mediterranean fever was established using the Tel-Hashomer criteria [1]. Blau syndrome was diagnosed on the basis of the characteristic clinical triad of granulomatous arthritis, dermatitis and uveitis occurring together with a NOD2 variant, following the original identification of NOD2 as the gene underlying the disease [2] and the subsequent clinical descriptions of the phenotype [3,4]. VEXAS syndrome was diagnosed according to the consensus guidance of the American College of Rheumatology, developed by the International VEXAS Working Group expert panel [5].

[1] Livneh A, et al. Criteria for the diagnosis of familial Mediterranean fever. Arthritis Rheum. 1997. doi:10.1002/art.1780401023. PMID:9336425.

[2] Miceli-Richard C, et al. CARD15 mutations in Blau syndrome. Nat Genet. 2001. doi:10.1038/ng720.

[3] Kaufman KP, et al. Distinguishing Blau Syndrome from Systemic Sarcoidosis. Curr Allergy Asthma Rep. 2021. doi:10.1007/s11882-021-00991-3. PMID:33560445.

[4] Wang OJE, et al. Blau Syndrome (Juvenile Systemic Granulomatosis): State-Of-The-Art Review. Pediatr Dermatol. 2025. doi:10.1111/pde.16001. PMID:40456554.

[5] Mekinian A, et al. American College of Rheumatology Guidance Statement for Diagnosis and Management of VEXAS Developed by the International VEXAS Working Group Expert Panel. Arthritis Rheumatol. 2025. doi:10.1002/art.43287.

Supplementary Tables

Supplementary Table S1. Detailed Genetic Profiles Analyzed in Table 3

| Gene | cDNA (reference transcript) | Protein | ClinVar classification |
| --- | --- | --- | --- |
| MEFV | NM_000243.3:c.605G>A | p.Arg202Gln | Benign/Likely benign |
| MEFV | NM_000243.3:c.329T>C | p.Leu110Pro | Conflicting classifications of pathogenicity |
| MEFV | NM_000243.3:c.442G>C | p.Glu148Gln | Conflicting classifications of pathogenicity |
| MEFV | NM_000243.3:c.1105C>T | p.Pro369Ser | Conflicting classifications of pathogenicity |
| MEFV | NM_000243.3:c.1223G>A | p.Arg408Gln | Conflicting classifications of pathogenicity |
| MEFV | NM_000243.3:c.1508C>G | p.Ser503Cys | Conflicting classifications of pathogenicity |
| NOD2 | NM_022162.3:c.931C>T | p.Arg311Trp | Conflicting classifications of pathogenicity |
| NOD2 | NM_022162.3:c.1411C>T | p.Arg471Cys | Benign/Likely benign |
| UBA1 | NM_003334.4:c.122T>C | p.Met41Thr | Conflicting classifications of pathogenicity |
| UBA1 | NM_003334.4:c.121A>G | p.Met41Val | Conflicting classifications of pathogenicity |

Supplementary Table S2. Association between dietary intake and spontaneous resolution of fever, adjusted individually for potential confounders.

| **Cohort** | **Adjusted for** | **Dietary intake OR (95% CI)** | **p** | **Covariate OR (95% CI)** | **p** |
| --- | --- | --- | --- | --- | --- |
| Full cohort (n = 116) | — (univariable) | 1.016 (1.001-1.031) | 0.039 | — | — |
| Full cohort (n = 116) | CRP (per 1 mg/dl) | 1.016 (1.001-1.031) | 0.04 | 1.002 (0.941-1.066) | 0.959 |
| Full cohort (n = 116) | Albumin (per 1 g/dl) | 1.013 (0.997-1.029) | 0.111 | 1.678 (0.838-3.359) | 0.144 |
| Full cohort (n = 116) | Hemoglobin (per 1 g/dl) | 1.012 (0.996-1.027) | 0.137 | 1.208 (0.982-1.485) | 0.073 |
| Full cohort (n = 116) | Abnormal renal function (yes vs no) | 1.012 (0.997-1.028) | 0.119 | 0.192 (0.042-0.886) | 0.034 |
| Excluding undiagnosed without resolution (n = 113) | — (univariable) | 1.017 (1.002-1.032) | 0.031 | — | — |
| Excluding undiagnosed without resolution (n = 113) | CRP (per 1 mg/dl) | 1.017 (1.002-1.033) | 0.03 | 1.010 (0.948-1.077) | 0.753 |
| Excluding undiagnosed without resolution (n = 113) | Albumin (per 1 g/dl) | 1.014 (0.998-1.030) | 0.09 | 1.558 (0.768-3.162) | 0.220 |
| Excluding undiagnosed without resolution (n = 113) | Hemoglobin (per 1 g/dl) | 1.013 (0.997-1.028) | 0.113 | 1.189 (0.967-1.463) | 0.101 |
| Excluding undiagnosed without resolution (n = 113) | Abnormal renal function (yes vs no) | 1.013 (0.998-1.029) | 0.097 | 0.183 (0.040-0.841) | 0.029 |

Each model contains dietary intake together with the single covariate shown. Odds ratios, 95% confidence intervals and p values were obtained by multivariable logistic regression. Dietary intake is the mean percentage of the served meal consumed during the first 3 days of hospitalisation. The odds ratios per 10% increment are 1.17 (univariable), 1.17 (adjusted for C-reactive protein), 1.13 (albumin), 1.12 (haemoglobin) and 1.13 (abnormal renal function) in the full cohort, and 1.18, 1.18, 1.15, 1.13 and 1.14 respectively in the restricted cohort. The restricted cohort excludes the 3 patients who remained undiagnosed without spontaneous resolution, so that the comparison is between the 25 patients who remained undiagnosed and resolved spontaneously and the 88 patients in whom a diagnosis was established.
